# Elevated Plasma Levels of CXCL13 Chemokine in Saudi Patients With Asthma Exacerbation

**DOI:** 10.7759/cureus.21142

**Published:** 2022-01-12

**Authors:** Wael Alturaiki

**Affiliations:** 1 Department of Medical Laboratory Sciences, College of Applied Medical Sciences, Majmaah University, Majmaah, SAU

**Keywords:** b cells, total ige, cxcr5, cxcl13, asthma

## Abstract

Background: Bronchial asthma is a lung disorder characterized by chronic allergic inflammation of the airways, and several of the immune and non-immune cells contribute to asthma's pathogenicity. B-cell activation plays an essential role in developing allergic inflammation in the lungs. CXCL13 is a potent B-cell chemoattractant* *chemokine, which has a crucial role in the recruitment and trafficking of B cells after interaction with its receptor CXCR5. This study is aimed to evaluate plasma levels of CXCL13 and its receptor CXCR5 in Saudi patients with asthma exacerbation relative to healthy controls.

Methods: A total of 23 patients with asthma exacerbation and 20 healthy controls participated in this study. Total immunoglobulin E (IgE) and CXCL13 protein levels were measured in the plasma of patients with asthma exacerbations and healthy controls by specific enzyme-linked immunosorbent assay (ELISA). Gene expression mRNA for CXCR5 was measured using real-time polymerase chain reaction (RT-PCR).

Results: Total IgE protein concentrations were elevated significantly in asthma exacerbation patients than that in healthy controls. CXCL13 protein levels were increased significantly in the asthma group relative to healthy controls. In addition, CXCR5 mRNA levels were elevated significantly in the asthma group than in the healthy controls.

Conclusions: Measurement of CXCL13 and CXCR5 may be used as an additional biomarker of asthma exacerbation, and targeting CXCL13 or its receptor may be used as new treatment options in asthma.

## Introduction

Bronchial asthma is a lung disorder characterized by chronic allergic inflammation in the airways with several symptoms including coughing, wheezing, airway smooth muscle construction, and deflects in breathing. Several immune and non-immune cells contribute to asthma's pathogenesis such as neutrophils, T lymphocytes, eosinophils, mast cells, airway epithelial cells, and other cellular components [1,2]. Also, asthma is considered one of the top chronic diseases in Saudi Arabia [3]. Asthma is of multiple types, and atopic asthma is the most common one mediated by immunoglobulin E (IgE) response [4]. B lymphocytes have an essential role in enhancing allergic inflammation in the airways, and it regulates the immune response in the lung through the production of Ig E antibody following the exposure of dendritic cells (DCs) to specific allergens that can be processed and presented as antigenic peptide and displayed to effector CD4+ T-helper lymphocytes (Th2) that are further enhancing allergic inflammation via production of cytokines, such as IL-4, IL-5, and IL-13 [4]. Furthermore, understating cytokines that regulate B-cell response in asthma is essential to know how B cells are activated, migrated, differentiated, and produce antibodies.

CXCL13 is a potent B-cell chemoattractant factor after interaction with its receptor (CXCR5) that is normally expressed at the surface of B cells and can facilitate B-cell migration and the follicle formation B-cell zones in the germinal center lymphoid tissues [5]. However, CXCL13 can be expressed in non-lymphoid tissues such as the lungs following respiratory viral infection and bronchial asthma [6-8].

The current study is designed to examine the airway inflammation in bronchial asthma by measuring the plasma levels of CXCL13 protein in patients with asthma exacerbation and to evaluate the expression of the CXCR5 receptor as a potential parameter to assess airway inflammation.

## Materials and methods

Subjects

Twenty-three people (13 males and 10 females, with a mean age of 33 years) were initially diagnosed with typical bronchial asthma based on Saudi infinitive guidelines [9] and admitted to emergency rooms at Al Zulfi General Hospital, Saudi Arabia, due to asthma exacerbation. All patients were examined by a respiratory consultant to assist their clinical status. Patients with apparent exacerbation symptoms such as wheezing, coughing, difficult breathing, speaking, chest tightness, persistence history with asthma, and required mechanical ventilation were selected. In addition, patients who were smoking or suffered from other pulmonary diseases, such as cystic fibrosis, chronic obstructive pulmonary disease (COPD), diabetes mellitus, blood hypertension, airway anomalies, cardiac disease, and any other chronic lung diseases, were excluded from the study. Besides, 20 subjects were enrolled as the healthy normal control group (13 males and seven females, with a mean age of 34 years). Control groups were healthy blood donors selected from the blood bank at the Al Zulfi General Hospital. Control groups were chosen based on the following criteria: no wheezing cases, no medical history of allergic diseases, no pregnancy, no obesity, no other chronic diseases, no infection within the past two weeks, no recent vaccination, and no smoking. All study subjects were obtained written informed consent, and the study was approved by the Ethical Committee at Majmaah University (Approval No.: MUREC-April.01/COM-206).

Separation of blood and collection of plasma and peripheral blood mononuclear cells

Five milliliters of peripheral blood (5 ml) was added to the anticoagulant ethylenediaminetetraacetic acid (EDTA) tube. Blood was separated using the Ficoll-Paque separation method (cat: GE17-5442-02, Sigma Aldrich, UK). Briefly, an equal volume of ficoll was added to the blood gently. After that, blood tubes were centrifuged at 1800 rpm for 30 minutes at room temperature (25°C). Following centrifugation, plasma was collected from the top layer, aspirated, and liquated into an Eppendorf tube; peripheral blood mononuclear cells (PBMCs) were isolated from the interface layer. After that, cells were re-suspended in Roswell Park Memorial Institute (RPMI) complete medium containing 12.5% fetal calf serum (FCS) and 20% dimethyl sulfoxide (DMSO) and stored at -20°C for gene expression.

Quantification of total IgE and CXCL13 protein by ELISA

Total IgE (Cat: ab108650, Abcam plc, Cambridge, UK) and CXCL13 chemokine (Cat: DY801, R&D, UK) levels were quantified in the plasma of the asthma group and healthy controls as described by the manufacturer. Protein concentrations were evaluated using KC Junior software (BioTek, Vermont, United States).

Isolation of RNA and measurement of CXCR5 mRNA by real-time polymerase chain reaction

RNeasy Mini Kit was used to extract RNA from the PBMCs according to the manufacturer's instructions (Cat: No./ID: 74104, QIAGEN, Manchester, UK). After that, cDNA was synthesized from RNA using reverse transcriptase enzyme and random primers (Cat: 4368814, Life Technologies, Thermo Fisher Scientific, UK). CXCR5 mRNA expression was then determined using Taqman real-time polymerase chain reaction (RT-PCR) probes (Cat: Hs00540548_s1, Life Technologies, Thermo Fisher Scientific, UK), and expression was normalized to the housekeeping gene L32 (cat: Hs00388301_m1, Life Technologies, Thermo Fisher Scientific, UK). Applied Biosystems™ 7500 Sequence Detection System (Thermo Fisher Scientific, UK) measured the gene mRNA expression.

Statistical analysis

The statistical analyses were performed using GraphPad Prism 6 software (GraphPad Software, San Diego, California). All the experimental data were assessed using the non-parametric Mann-Whitney U test, and the results were presented as mean ± standard deviation (SD). P-values < 0.05 were considered significant.

## Results

Asthma patients with elevated significant levels of total IgE

**Figure 1:**
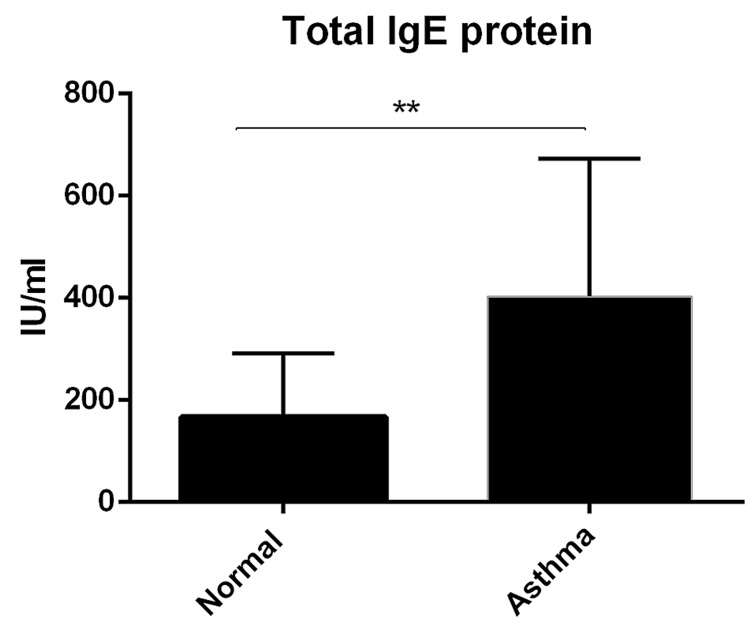
Quantification of total IgE protein concentration IgE protein concentration was determined in the plasma by ELISA assay. Data was presented as mean ± SD (normal = 20, asthma = 23, P < 0.002). ELISA, Enzyme-linked immunoassay.

Atopic or allergic asthma is a kind of asthma characterized by an IgE response. To further validate the allergic status of patients with asthma, total IgE was evaluated in the plasma. Total IgE levels in plasma were elevated significantly in the asthma group (mean 400 Iu/ml) than in the normal controls (167 Iu/ml, P < 0.002) (Figure 1).

Expression of CXCL13 chemokine levels in patients with asthma exacerbation

**Figure 2:**
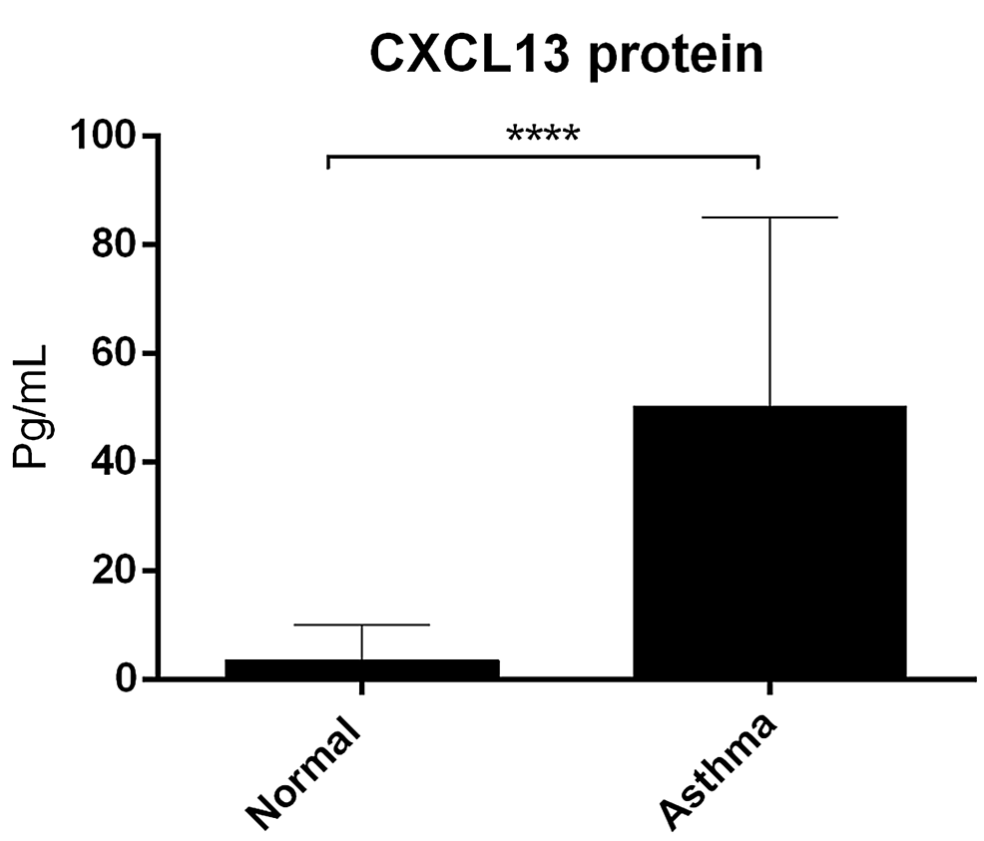
Quantification of CXCL13 protein concentration CXCL13 protein concentration was determined in the plasma by ELISA assay. Data was presented as mean ± SD (normal = 20, asthma = 23, P < 0.0001). ELISA, Enzyme-linked immunoassay.

CXCL13 chemokine levels were increased significantly in the asthma group (mean 50 pg/ml) in comparison to normal controls (3.6 pg/ml, P < 0.0001) (Figure 2).

CXCR5 mRNA is increased significantly in patients with asthma

**Figure 3:**
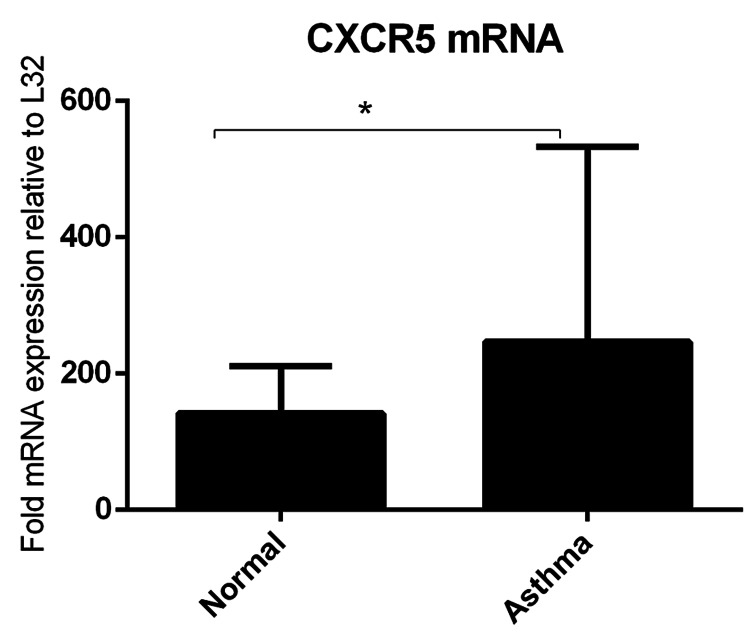
RT-PCR analysis of CXCR5 Expressions of CXCR5 mRNA levels in asthma patients relative to healthy controls. Data were presented as mean ± SD (normal = 5, asthma = 7, P < 0.05). RT-PCR, Real-time polymerase chain reaction.

RT-PCR analysis of PBMCs isolated from the asthma group and healthy controls showed a significant increase in the expression of CXCR5 mRNA of patients asthma (mean = 246 fold expression, P < 0.05) relative to normal controls (mean = 141) (Figure 3).

## Discussion

This study was carried out to assess the plasma levels of CXCL13 chemokine and its receptor CXCR5 as potential biomarkers in asthma exacerbation. Atopic asthma is featured with increased IgE levels [10]. In line with this, total IgE levels were increased significantly compared to the healthy controls (Figure 1). CXCLl3 chemokine mediates an essential role in B-cell response and B-cell follicle formation in the lymphoid tissues' germinal centers (GC). Increased expression of CXCL13 was found in several types of airway diseases, such as COPD [11] and lung cancer [12]. In addition, expression of CXCL13 was found in other non-lung disorders such as rheumatoid arthritis and multiple sclerosis [13,14], which further confirmed that CXCL13 is contributed to the development of chronic inflammatory diseases.

Several studies have instigated CXCL13 in asthma. It has been reported that CXCL13 levels in bronchoalveolar lavage fluid (BALF) fluid were elevated significantly in asthma patients relative to normal controls. When mice were challenged with ovalbumin, it resulted in upregulation expression of the CXCL13/CXCR5 levels that are correlated with the recruitment of B cells and CD4 T cells, airway inflammation, and formation of bronchial-associated lymphoid tissue compared to the control animals. Targeting of ovalbumin-sensitized mice with anti-CXCL13 resulted in a reduction in the migration of cells, airway inflammation, and decreased development of bronchial-associated lymphoid tissue formation [8]. Consistently, it has been found that B-cell follicles were observed in bronchial lung tissue sections from patients with asthma and increased significantly at the severity of asthma relative to healthy controls, and increased B cells were positively correlated with increased expression of CXCL13 [15].

Furthermore, CXCL13 levels in the asthmatic children's sputum were significantly higher than those in the control group. Increased expression of CXCL13 was positively associated with sputum eosinophils and eosinophil cationic protein (ECP), demonstrating that CXCL13 plays an important role in the pathophysiology of childhood asthma and the possibility for use as additional parameters in asthma diagnosis [16].

Moreover, in the current study, CXCL13 levels in plasma were increased significantly in patients with exacerbation relative to healthy controls (Figure 2), and this finding is in agreement with one study that reported CXCL13 levels in serum were higher in acute asthmatic patients than in chronic asthmatic patients [17]. Collectively, this suggests that CXCL13 may be used as a potential maker to evaluate acute exacerbation of asthma. Additionally, CXCR5 mRNA levels were higher in asthma exacerbation patients compared to healthy controls (Figure 3). CXCR5 can also be expressed by follicular helper T (TFH) cells subset CXCR5+CD4+ T and played an essential role in enhancing IgE production in asthma [18]. Taken together, this finding suggests that CXCR5 may reduce IgE production and, therefore, can be useful for asthma treatment.

The current study's limitations are summarized as follows: (1) a subsequent large study among patients with different asthma severities may shed light on the exact expression of CXCL13 chemokine and its receptor CXCR5 in asthma. (2) Measuring CXCL13 in lung secretions may reflect the status of allergic inflammation of the airways. Thus, further studies should examine whether lung chemokine expression is a useful progression biomarker of asthma activity.

## Conclusions

In summary, measurement of CXCL13 levels in plasma may provide diagnostic parameters for assessing airway inflammation and monitoring asthma exacerbation. Besides, measuring CXCR5 may also indicate activation of B cells and TFH, which can further enhance IgE production and allergic inflammation. Collectively, targeting CXCL13 or blocking CXCR5 may contribute to asthma treatment and reduce the severity of asthma.
